# A Reliability Study of the Load Distribution Percentage While Walking Using Curalgia Feet Sx Smart Insoles

**DOI:** 10.7759/cureus.68232

**Published:** 2024-08-30

**Authors:** Deepjyoti Das, Maitri Chaturvedi, Maneesh Arora, Sukanya Dikshit, Vishwal Padole

**Affiliations:** 1 Physiotherapy, Sardar Bhagwan Singh University, Dehradun, IND; 2 Incubation Center, Shriram Institute for Industrial Research, Gurugram, IND; 3 Founder, WeRehab Technologies Pvt. Ltd., Nagpur, IND; 4 Incubation Center, Shriram institute for Industrial Research, Gurugram, IND; 5 Co-Founder, WeRehab Technologies Pvt. Ltd., Nagpur, IND

**Keywords:** interrater reliability, foot and ankle biomechanics, foot, smart insoles, gait training, gait analysis

## Abstract

Background

Gait analysis has evolved through many years of research. Many methods are used to analyze the gait of a subject. Recent times have shown a high demand for wearable sensor-based insoles integrated with smartphone-based devices used for gait analysis due to ease of use. This study utilized Curalgia Feet Sx Smart Insoles and its software toolset, Gait Analysis+, designed and manufactured in India making it an accessible and cost-effective option. The Curalgia Feet Sx Smart Insoles allow for a broad range of biofeedback-based rehabilitation and recovery training for several patients and have many applications, such as sports performance enhancement and neurological disorder rehab (e.g., brain stroke rehab). The system also significantly delays the onset of neurodegenerative illnesses by providing balance and proprioceptive training. The smart insole can help the athlete, the coach, and the sports medicine team get the on-field data in real-time, which will help them understand if any technical or biomechanical alterations are required. This may help in performance enhancement. This study aimed to determine the interrater reliability of the load distribution percentage parameter of the Curalgia Feet Sx Smart Insole for both feet while walking in a controlled setting.

Methodology

A total of 120 subjects were enrolled in the study. In total, 90 subjects were randomly selected using Research Randomizer which included male and female students and staff at Sardar Bhagwan Singh University. The subjects were asked to come to the research lab of the physiotherapy department wearing their sports shoes. Curalgia Feet Sx insoles were inserted into the shoe firmly to fit properly. Two assessors took two readings after the smart insole was connected to the smartphone-based application, GaitAnalysis+, via Bluetooth. The dynamic analysis option was selected, and each subject’s analysis was done one after another with a desirable break in between. Each subject walked for three minutes at their normal speed after pressing “Start Analysis.” At the three-minute mark, the subjects were asked to press “Stop Analysis” and the investigator downloaded the report on the smartphone. The data collected was compiled as the cumulative weight in kg (load distribution) borne and the % weight (load distribution %) borne by each foot for the duration of the walk. Statistical analysis was done using Karl Pearson’s test and interclass correlation calculation.

Results

Assessor 1 and Assessor 2 collected readings for the left foot as “L” and the right foot as “R.” Assessor 1 readings were L1-R1 for load distribution and L1% and R1% for load distribution %. Assessor 2 readings were L2-R2 for load distribution and L2% and R2% for load distribution %. The r value (correlation coefficient) was calculated using the load distribution. The mean value of L1 was 337.46 (SD=94.16). The mean L2 was 313.6 (SD=104.40). The R1 mean was 229.03 (SD=112.88), and the R2 mean was 233.011 (SD=79.84). The r was 0.7171 for the left foot and 0.7502 for the right foot, suggesting an excellent correlation. The ICC was calculated for load distribution %. The means of L1% was 55.94, L2% was 57.59, R1% was 44.06, and R2% was 42.41. The ICC was found to be 0.91 for both feet, suggesting high interrater reliability for the tested parameter.

Conclusions

The findings confirmed that the Curalgia Feet Sx Smart Insoles presented good interrater reliability for the load distribution % parameter.

## Introduction

Gait analysis has been extensively employed in numerous contexts, including sports, rehabilitation, and medical diagnostics. It is also utilized in orthopedics and rehabilitation to monitor patients following surgery and is particularly helpful in applications that require orthopedic assistive devices [1]. Gait analysis has evolved through many years of research. Many methods are used to analyze the gait of a subject. Sensor-based insoles are one of the methods used for gait analysis. Moreover, there has been a great demand for wearable sensors integrated with smartphone-based devices used for gait analysis [2]. The Curalgia Feet Sx Smart Insoles and a software toolkit enable a wide variety of biofeedback-based rehabilitation and recovery training for many patients and have several applications, including brain stroke rehab and sports performance improvement. The system also serves as an important tool in delaying the onset of neurodegenerative diseases. For athletes, using this smart insole, the coach and the sports medicine team can obtain the athlete’s on-field data, which will help them on the biomechanical level. This data will be used to implement training methods to reduce injury risk and fatigue and improve athletic performance.

A typical gait analysis is mainly visual, observing a patient as they walk [3]. Although it is a crucial assessment component, this section lacks objective information on the center of force (CoF), step time, swing time, stride length, force, and weight distribution. Information like this is important for the diagnosis and treatment of gait issues, but it cannot be accurately obtained through visual analysis. Here the Curalgia gait analysis tool comes in. These in-shoe sensors add objective, repeatable, and actionable data to the evaluation process. This product has been conceptualized and manufactured in India, making it easily accessible and cost-efficient. The insoles analyze gait using a multisensor system and record various parameters, viz., load distribution percentage, step count, cadence, stride length, ground contact time, gait phase analysis, foot pronation, strike distribution, overstride, and body balance. However, no research has been published to date on the reliability of this product. Evaluation of the product’s reliability plays a very important role, as it helps in determining the consistency, reproducibility, sensitivity, and accuracy of the product. This is crucial to ensure that the data is sound and replicable and that the results are accurate. To ensure the integrity and quality of a measurement tool, evidence of reliability is required.

Determining the interrater reliability indicates how closely and accurately the study’s data reflect the measured variables, making it a crucial study. A few studies [3-6] have been conducted to evaluate the reliability of various smart insoles available on the market; however, the companies producing these products are based outside of India. Given that Curalgia Foot Sx is among the first smart insoles to be developed in India and that no studies have been released on it, it is necessary to investigate the product’s dependability. Additionally, taking assessments requires a lot of work and is cumbersome with the current gold-standard technology. The smart insoles are portable, easy to use, and cost-effective and will help make the assessments easier. This study determined the reliability of the load distribution percentage (%) parameter of the insole system as the nature of foot mechanics and the specific areas of the foot that bear the body weight while ambulation play a vital role in having a normal gait. It is also helpful in preventing injuries related to balance, faulty foot positioning, faulty force distribution, etc.

## Materials and methods

Study sample

A total of 120 subjects were enrolled in the study. Of these 120 subjects, 90 were recruited by single-blinded randomization done by Research Randomizer, an online tool that helps generate random numbers for experimental conditions. The selected candidates were recruited in the study after they provided written consent. For this study, the subjects were recruited irrespective of their gender. To qualify for the study, participants could not have had any known gait abnormalities, recent fractures, or deformities, and their shoe sizes needed to be UK 6, 7, and 8. Their participation was voluntary. The participants were recruited from Sardar Bhagwan Singh University, Dehradun.

Experimental protocol

The subjects were brought to the research lab at the Department of Physiotherapy wearing their sports shoes. The smart insoles (Curalgia Feet Sx) were inserted inside the shoes very firmly to fit properly, according to their shoe size. During the procedure, the subjects were instructed not to wear socks. After the subject wore the shoe, the smart insole was connected to the smartphone-based application, GaitAnalysis+, via Bluetooth connectivity. Two assessors took two readings for each subject on their smart devices. As the insole connected to the app, the dynamic analysis option was selected. After the “Start Analysis” icon was pressed, the subject walked for three minutes at their normal speed. The manufacturers suggest subjects walk for at least three minutes for better results, which was found in their work during the prototype stage and is mentioned in the footnotes of the instruction manual of the manufacturer. The smart device was kept in the connectivity range of the insole while the recording was under process. The subjects were asked to walk on the tiled surface of the research lab. After the three-minute mark, the “Stop Analysis” icon was pressed so that the investigator could download the report on the smartphone itself.

Instrumentation

Curalgia Feet Sx Smart Insoles

**Figure 1:**
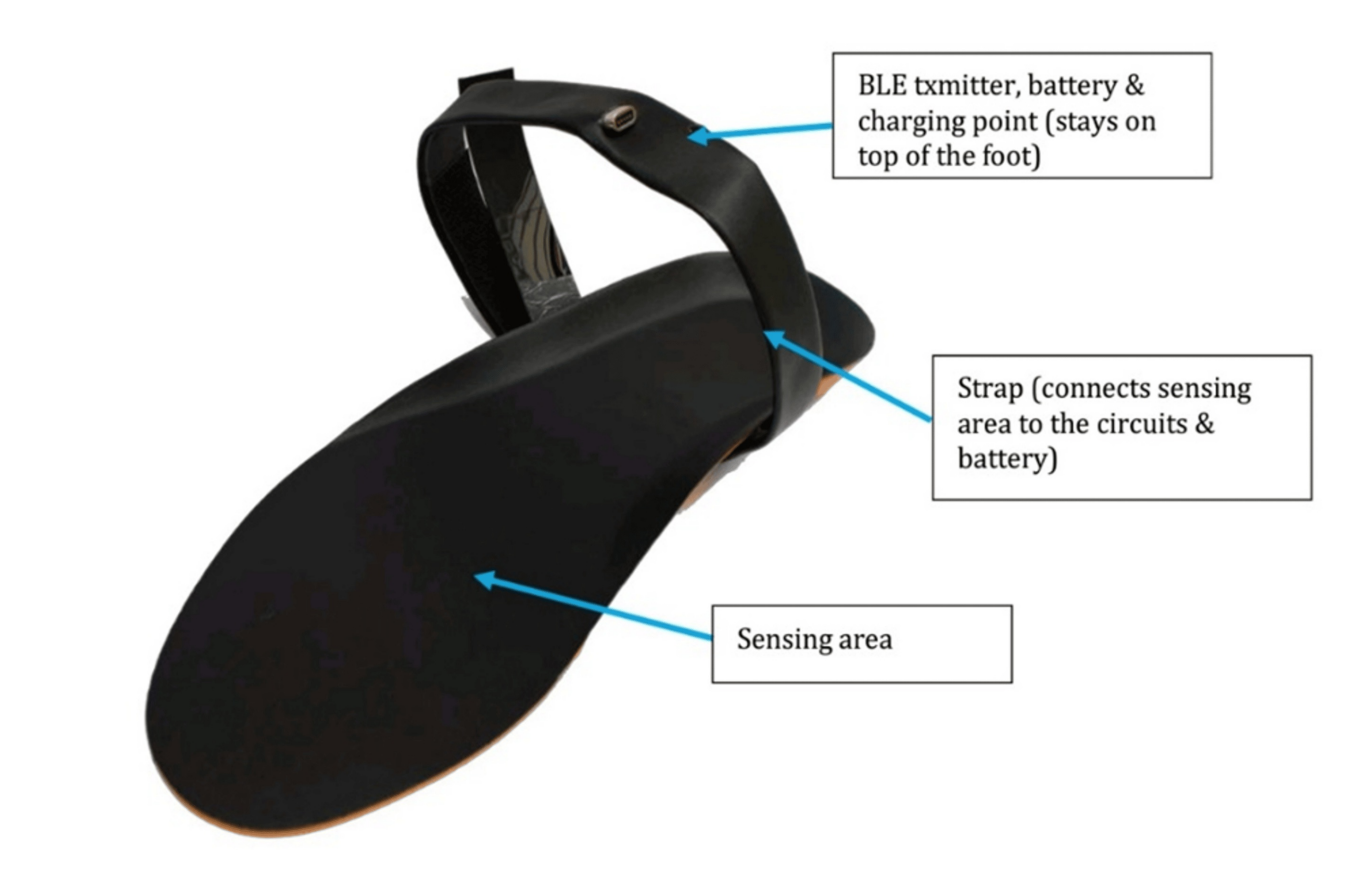
Structure of Curalgia Feet Sx Smart Insole (INSIGHT variant). The Curalgia Feet Sx Smart Insole shown in the image marks the strap of the insoles which keeps it stable while analysis is in progress. The strap has circuitry embedded in it and the top section has a BLE transmitter, the battery, and the charging port. The sole area is embedded with an array of sensors that record various parameters. Picture credit: WeRehab Technologies Pvt. Ltd. (added with permission).

When combined with a software toolset, the Curalgia Foot Sx (Figure 1) smart insoles allow for a broad range of biofeedback-based rehabilitation and recovery training for several patients and applications, such as sports performance enhancement and brain stroke rehab. Additionally, the system may play a significant role in delaying the onset of neurodegenerative illnesses by providing balance and proprioceptive training, which can be studied separately. It can be used as a valuable tool in the assessment of gait parameters. It can record walking data in real-time while performing activities of daily living through the sensors embedded in the insole. These sensors are placed at points that are crucial to accurately perform posture and locomotion analysis. The smart insole is paired with a smartphone-based application, GaitAnalysis+, via Bluetooth connectivity. After analysis, parameters such as the number of steps and load distribution are recorded in the application.

Statistical analysis

The reliability of the load distribution percentage of the Curalgia Feet Sx Smart Insole was examined using the collected data. Data and statistical analysis were performed using SPSS version 20.0 (IBM Corp., Armonk, NY, USA) and MS Excel (Microsoft Corp., Redmond, WA, USA). Karl Pearson’s test and interclass correlation (ICC) calculation were done to find the statistical significance of the compiled data.

## Results

**Figure 2:**
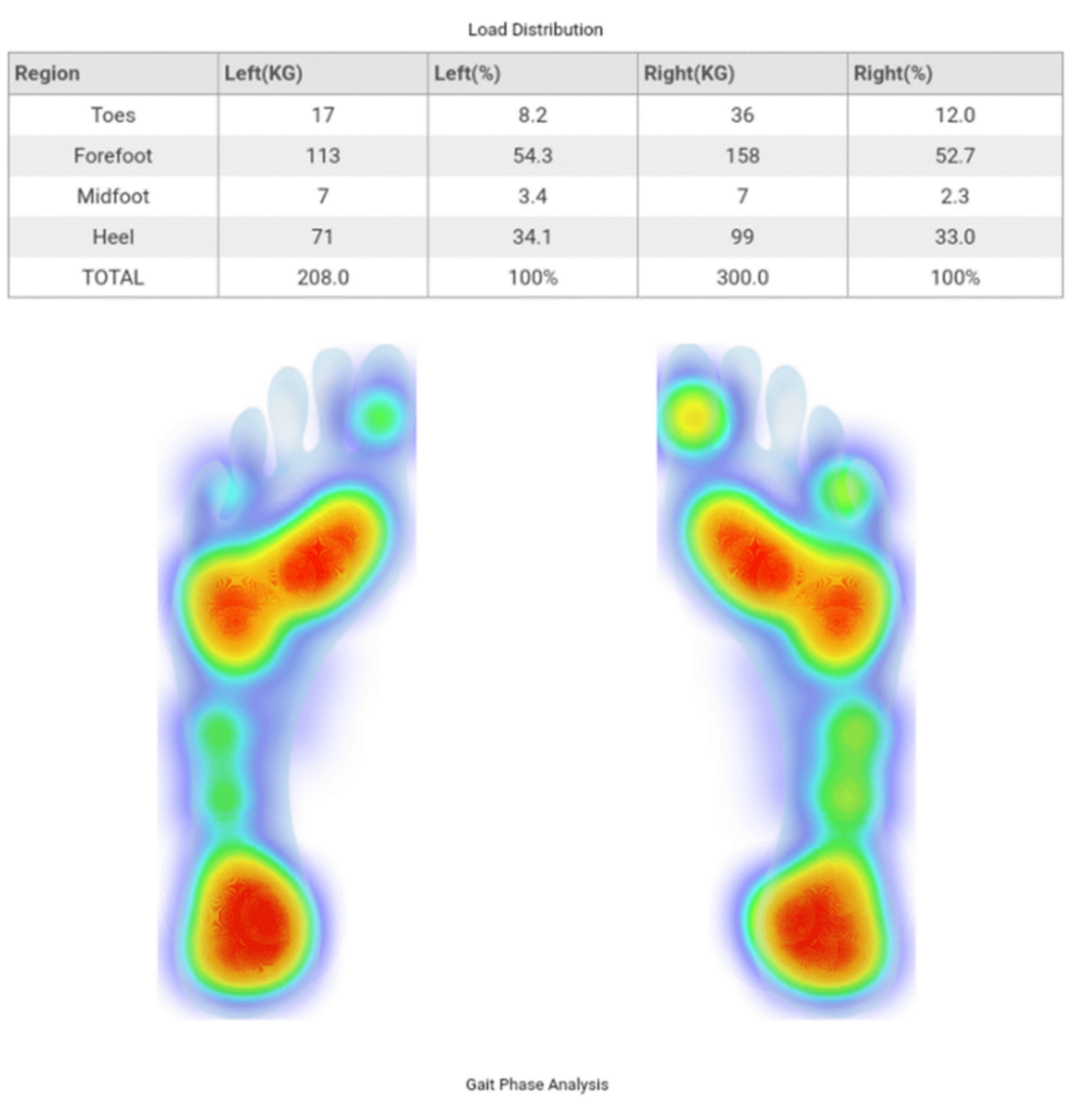
Automated analysis report generated by GaitAnalysis+. The image is a sample of the load distribution parameter recorded by the smart insoles for one subject. The table within the image shows how the insole-app interaction calculates the load distribution in kg and %. The foot image shows weight-bearing areas within the foot as recorded by the insole-app interaction.

The load distribution data was calculated by GaitAnalysis+ using both quantitative value and a real-time image generator as shown below (Figure 2). The figure shows that the insole-app interaction records and presents the load distribution data in kg/square inch and %. The table within the image shows the load distribution in kg, the cumulative weight borne by the foot of one side for the total duration the subject walked. The percentage (%) shows the % weight borne by a specific area of the foot during the complete walk duration. The heat map in the figure shows the areas that bear the weight, where the red region bears the most weight and light blue bears the minimum weight.

Two different assessors (further mentioned as Assessor 1 and Assessor 2) collected readings for the left foot, denoted as “L,” and the right foot, denoted as “R.” The readings collected by Assessor 1 were designated as L1 and R1 for load distribution in kg and L1% and R1% for load distribution percentage. Whereas, the readings collected by Assessor 2 were designated as L2 and R2 for load distribution in kg and L2% and R2% for load distribution percentage.

The correlation coefficient r was calculated by the load distribution (kg). For the left foot, the mean value of L1 was 337.46 (SD= 94.16). The mean value for L2 was 313.6 (SD = 104.40). Later, the r-value, i.e., Karl Pearson’s correlation coefficient was calculated for the left foot and was found to be 0.7171 (Table 1). Similarly, the values for the right foot were also calculated. The mean R1 value was 229.03 (SD = 112.88), and the mean R2 value was 233.011 (SD = 79.84). Karl Pearson’s correlation coefficient was calculated for the right foot and was found to be 0.7502 (Table 1).

**Table 1:** Values of load distribution in kilograms. The table shows the mean (mean cumulative weight borne by the feet in kg/square inch), SD, and Karl Pearson’s correlation coefficient by Assessor 1 and Assessor 2. L1 = Assessor 1 left foot reading; L2 = Assessor 2 left foot reading; R1 = Assessor 1 right foot reading; R2 = Assessor 2 right foot reading. SD = standard deviation; r = Karl Pearson’s correlation coefficient

Foot		Mean (kg/square inch)	SD	r
Left	L1	337.46	94.16	0.7171
	L2	313.6	104.40	
Right	R1	229.03	112.88	0.7502
	R2	233.011	79.84	

The r value was found to be 0.7171 for the left foot and 0.7502 for the right foot. As the r value for both feet was >0.7, we can consider it an excellent correlation between the data analyzed.

To find the interrater reliability, ICC was calculated using the load distribution percentage data. Here, load distribution percentage means the % weight borne by each foot for the duration the subject walked, where L1% and R1% are the readings of Assessor 1 and L2% and R2% are the readings by Assessor 2, with L denoting the left foot and R denoting the right foot.

As shown in Table 2, the L1% was 55.94%, and the mean L2% was 57.59%. Calculating this data, we found that the ICC value for the left foot was 0.91. Similarly, the mean R1% was 44.06%, and the mean R2% was 42.41%. The ICC value for the right foot was 0.91. Since the ICC value is very close to 1, it suggests high interrater reliability of the load distribution percentage while walking using the Curalgia Feet Sx Smart Insoles.

**Table 2:** The readings of load distribution % during the three-minute walk. L1% = load distribution % at the left foot by Assessor 1; R1% = load distribution % at the right foot by Assessor 1; L2% = load distribution % at the left foot by Assessor 2; R2% = load distribution % at the right foot by Assessor 2. Min = minimum; Max = maximum; SD = standard deviation; ICC = interclass correlation

	Assessor	Min-Max	Mean (%)	SD	ICC
L1%	1	35.53-78.45	55.94	12.41	0.91
L2%	2	33.65-79.88	57.59	12.92	
R1%	1	21.55-64.47	44.06	12.41	0.91
R2%	2	20.12- 66.35	42.41	12.92	

## Discussion

This study aimed to examine the interrater reliability of the load distribution percentage parameter of Curalgia Feet Sx, a newly introduced smart insole for gait analysis. The load distribution percentage data of 90 subjects were collected. Each participant performed two walking trials, and ICC calculation and Karl Pearson’s test were applied to the collected data to calculate the correlation between the two walking trials.

As a single insole size cannot cover multiple shoe sizes, insole coverage underneath each foot will vary. Hence, foot size needs to be checked before enrolling subjects. The inclusion in the study was limited to subjects with shoe sizes UK 6, 7, and 8 as the available insole sizes are UK 6, 7, and 8. Potential sources that could create variation in the data were removed by ensuring conditions, including standardized footwear and the use of a predefined walk time (three minutes). However many factors cannot be excluded such as vertical ground reaction force (vGRF) as researchers have suggested that there is little difference between males and females in their vGRF during running and walking as vGRF parameters depend upon speed, ground contact, etc. [4].

Research has stated a need to create a special environment for the assessment while using small variable devices linked to phones, and tablets for pedestrian evaluation, and these devices still need to be further studied in the future [5]. However, piezo-resistive force sensors and six-axis inertial motion sensors which work at 8 Hz were used within 4 mm foam on the toe and 5 mm on the heel giving the bending up to 50 mm to the insole to reduce external error-creating factors. The insole is 50 g per pair making it easy to carry. The 8 Hz frequency is more than sufficient for gait analysis as it had been mentioned that 5 Hz is sufficient for gait analysis. The six-axis accelerometer gives better support, that is, more than +6 g, and better dynamic analysis because the three-axis accelerometer can support up to +6 g and can be used conventionally for dynamic gait analysis [7,8]. As traditional gait analysis system has many limitations due to their high cost, installment, sophisticated environment, etc. [9], smart insoles are a great alternative as they are lightweight, slim in design, cost-effective, and cheaper than the traditional ones [5]. Insoles equipped with miniaturized embedded sensors are a potential solution for monitoring daily activities and foot posture, given that every individual wears shoes for hours a day and the present microelectromechanical system has made instrumentation less expensive [6].

Several researchers have proven the reliability of these smart insoles for analyzing gait [3-6]. Our study also shows the reliability of Curalgia Feet Sx with an r-value of 0.7171 in the left limb and 0.7502 in the right limb, with an r-value >0.7 having an excellent correlation. The ICC value was found to be 0.91, suggesting high interrater reliability of the tested parameter.

Other studies also used the test-retest reliability method to find the reliability in smart insoles where they found reliability ICC >0.910, indicating a good performance of measuring wearable insole pressure system (WIPS)-based gait parameters for distinguishing safety hazards. The study also supported the relevance of WIPS for the evaluation of safety hazards on construction sites [3,4,9-11].

A study also used the Wearable Gait Lab system which provided quantitative statistical support for balance tests, in which a wearable underfoot force sensing unit was used to record foot motions and plantar pressure data. The study showed that the unit was informative and reliable for determining the balance status of the subject [12]. Researchers tested the mechanical reliability for prolonged wear of an insole-based wearable sensor (SmartStep), which suggested that the SmartStep pressure sensors tolerated prolonged human wear [11]. Hence, these smart insoles can be used for assessing different foot abnormalities such as excessive foot pronation, excessive foot supination, and limb length discrepancy [13].

This study determined the interrater reliability of the load distribution % parameter. Future can be conducted to determine interrater reliability and reproducibility which includes testing all other parameters too. As the insoles are of fixed size, UK 6, 7, and 8, even the slightest variation in shoe size caused the insoles to slip into the shoe. The slip was not significant enough to disturb the readings but made walking slightly uncomfortable for the patient. Some patients did become conscious of their gait while testing. Although it did not change the results, it may become a possible obstacle when the insole is being used in a certain condition or being used for testing in patients.

## Conclusions

The study showed statistically significant results. We can conclude that the percentage of load distribution parameter in the Curalgia Feet Sx Smart Insole shows high interrater reliability and can provide reproducible and accurate results.
